# High-Throughput Drug Screening of Clear Cell Ovarian Cancer Organoids Reveals Vulnerability to Proteasome Inhibitors and Dinaciclib and Identifies AGR2 as a Therapeutic Target

**DOI:** 10.1158/2767-9764.CRC-25-0024

**Published:** 2025-06-25

**Authors:** Takuma Yoshimura, Takashi Kamatani, Aki Ookubo, Mio Takahashi, Manabu Itoh, Toshiki Ebisudani, Yohei Masugi, Tomomi Toyonaga, Junko Hamamoto, Keiko Saotome, Kensuke Sakai, Tomoko Yoshihama, Nobuko Moritoki, Shinsuke Shibata, Hiroyuki Yasuda, Toshiro Sato, Taka-Aki Sato, Daisuke Aoki, Wataru Yamagami, Tatsuhiko Tsunoda, Tatsuyuki Chiyoda

**Affiliations:** 1 Department of Obstetrics and Gynecology, Keio University School of Medicine, Tokyo, Japan.; 2 JSR-Keio University Medical and Chemical Innovation Center (JKiC), Keio University School of Medicine, Tokyo, Japan.; 3 Laboratory for Medical Science Mathematics, Department of Biological Sciences, School of Science, The University of Tokyo, Tokyo, Japan.; 4 Department of AI Technology Development, M&D Data Science Center, Institute of Integrated Research, Institute of Science Tokyo, Tokyo, Japan.; 5 Department of Precision Cancer Medicine, Center for Innovative Cancer Treatment, Institute of Science Tokyo Hospital, Tokyo, Japan.; 6 Division of Pulmonary Medicine, Department of Medicine, Keio University School of Medicine, Tokyo, Japan.; 7 JSR-Keio University Medical and Chemical Innovation Center (JKiC), Tokyo, Japan.; 8 Department of Pathology, Keio University School of Medicine, Tokyo, Japan.; 9 Electron Microscope Laboratory, Keio University School of Medicine, Tokyo, Japan.; 10 Department of Integrated Medicine and Biochemistry, Keio University School of Medicine, Tokyo, Japan.; 11 Research and Development Center for Precision Medicine, University of Tsukuba, Ibaraki, Japan.; 12 Laboratory for Medical Science Mathematics, Department of Computational Biology and Medical Sciences, Graduate School of Frontier Sciences, The University of Tokyo, Tokyo, Japan.; 13 Laboratory for Medical Science Mathematics, RIKEN Center for Integrative Medical Sciences, Yokohama, Japan.

## Abstract

There are currently no effective treatments available for clear cell ovarian cancer (CCC). In this study, we aimed to identify effective drugs for CCC through high-throughput drug screening (HTDS) using ovarian cancer organoids and determine novel therapeutic targets based on the biological characteristics of CCC through omics analysis. An ovarian cancer organoid biobank was established, and HTDS was conducted using CCC organoids based on libraries of 361 and 4,560 compounds. The efficacy of the identified drugs was verified in *in vitro* and *in vivo* experiments using a patient-derived organoid xenograft mouse model. Transcriptome analysis was performed to identify genes related to the pathways targeted by the identified drugs in CCC and to assess their potential as therapeutic targets. Proteasome inhibitors and dinaciclib were extracted using HTDS and shown to inhibit tumorigenesis *in vitro* and *in vivo*. CCC, like multiple myeloma, exhibited activated endoplasmic reticulum (ER) stress and unfolded protein response (UPR), and treatment with proteasome inhibitors further enhanced ER stress and UPR, ultimately leading to cell death. Transcriptome analysis identified *anterior gradient-2* (*AGR2*) as a key gene involved in UPR in CCC. CRISPR knockout of *AGR2* suppressed cell proliferation, increased sensitivity to proteasome inhibitors, and reversed platinum resistance in CCC. *AGR2* knockout also upregulated *Schlafen 11*, contributing to platinum sensitivity. ER stress and the UPR are activated in CCC, and proteasome inhibitors disrupt this balance, ultimately leading to cell death. AGR2 may serve as a potential therapeutic target in CCC.

**Significance:**

Proteasome inhibitors and dinaciclib are identified as effective drugs for CCC. CCC has a high basal UPR, and proteasome inhibition may disrupt this balance. AGR2 is involved in the UPR of CCC, and inhibiting AGR2 further enhances the UPR and confers platinum sensitivity, making it a potential therapeutic target.

## Introduction

Clear cell ovarian cancer (CCC) is a relatively rare subtype of ovarian cancer, accounting for approximately 25% of cases in Japan and 10% in other countries (1). CCC typically exhibits platinum resistance, with a reported response rate of 11.1% compared with 72.5% for serous carcinoma (2). Despite this resistance, CCC is often treated with debulking surgery and systemic platinum combination chemotherapy, similar to other histologic subtypes such as high-grade serous cancer (HGSC) and endometrioid ovarian cancer. Therefore, the prognosis for advanced or recurrent CCC is poor, highlighting the need for innovative therapeutic approaches.

The most common gene variants in CCC include *PIK3CA* and *ARID1A*, which are present in almost half of cases (3). *PIK3CA* is considered a druggable target, and novel phosphatidylinositol-3 kinase inhibitors are currently under investigation for CCC. On the other hand, *ARID1A* is not yet considered clinically druggable. However, research is ongoing on the use of *ataxia* telangiectasia and Rad3-related protein inhibitor ceralasertib and immune checkpoint inhibitors for CCC with *ARID1A* mutations. *ARID1A* mutations have been associated with high levels of tumor-infiltrating lymphocytes and PD-L1 expression in CCC (4), but it is still debated whether *ARID1A* mutations can serve as a biomarker for immune checkpoint inhibitor treatment (5). CCC is believed to originate from endometriosis, but *ARID1A* or *PIK3CA* mutations have been found in benign endometriosis and even in normal uterine endometrial glands (6, 7). Therefore, targeting drugs other than *ARID1A* and *PIK3CA* may be more beneficial in treating CCC.

We have developed an efficient method for culturing ovarian cancer organoids and established an ovarian cancer organoid biobank. Organoids exhibit genomic and phenotypic characteristics similar to their original tumors and demonstrate therapeutic sensitivity reflective of the clinical course (8). Although drug screening has typically been conducted using cancer cell lines in two-dimensional culture systems, differences in drug sensitivity between two-dimensional and three-dimensional (3D) culture systems have been observed (9), with 3D systems more accurately reflecting the clinical response (10). In this study, we conducted high-throughput drug screening (HTDS) using 361 and 4,560 drugs in our CCC organoid biobank. Furthermore, a novel therapeutic target for CCC was investigated based on the results of HTDS combined with omics analysis.

## Materials and Methods

### Chemicals and antibodies

The inhibitor library used in this study was the L1700 Bioactive Compound Library (Selleck Chemicals), containing 4,560 compounds, along with SCADS inhibitor kits (361 compounds, JSPS) for HTDS. Specific compounds such as YM155, staurosporine, ouabain, bortezomib, dinaciclib, carfilzomib, ixazomib, carboplatin, and paclitaxel were purchased from Selleck Chemicals. Additionally, cyclin-dependent kinase 2/9 (Cdk2/9) inhibitors and cucurbitacin I were purchased from Merck KGaA, whereas brefeldin A was purchased from Fujifilm Wako. Doxorubicin was purchased from Sigma-Aldrich. Antibodies used in the study, including anti–inositol-requiring enzyme 1α (IRE1α; #3294), anti-XBP1s (#40435), anti–protein kinase R–like ER kinase (PERK; #3192), anti–activating transcription factor 6 (ATF6; #65880), anti-ATF4 (#11815), anti-CHOP (#2895), anti-eIF2α (#5324), anti–phospho-eIF2α (#3398), anti–Bcl-2 (#4223), anti–cleaved caspase3 (#9664), anti–anterior gradient-2 (AGR2; #13062), anti–Schlafen family member 11 (SLFN11; #34858), and mouse and rabbit secondary antibodies (#7076, #7074), were purchased from Cell Signaling Technology. Furthermore, anti–β-actin (M177-3) was purchased from MBL.

### Public data resource

To validate the genetic mutations in the organoid samples and ensure their consistency with known mutations in CCC, whole-exome sequencing (WES) data from CCC were utilized as a validation dataset. These specimens were acquired and analyzed by the National Bioscience Database Center (RRID: SCR_000814) via the public database JGAS000076.

To assess whether the gene patterns in CCC organoid samples are enriched in the CCC signature similar to tissue CCC and to compare their enrichment levels with HGSC organoid and tissue samples, as well as normal ovarian expression data, we obtained multiple myeloma and HGSC samples from The Cancer Genome Atlas (TCGA) project (11) through the UCSC Xena platform (http://xenabrowser.net/datapages/, RRID: SCR_018938). Additionally, mRNA sequencing results of CCC, HGSC, and normal ovary samples were obtained from a published article (12), and normal ovarian data were sourced from the Genotype-Tissue Expression project (13).

### Gene expression analyses

RNA was extracted from the organoid samples using the RNeasy Micro Kit (Qiagen) following the manufacturer’s protocol. Subsequently, sequencing libraries were prepared using the NEBNext Ultra II Directional RNA Library Prep Kit for Illumina (New England Biolabs), MGIEasy RNA Directional Library Prep Set V2.0 (MGI Tech), or VAHTS Universal V8 RNA-seq Library Prep Kit for Illumina (Vazyme) for RNA sequencing. The RNA was then sequenced on the 150-bp paired-end read HiSeq X Ten (RRID: SCR_016385), NovaSeq 6000 (RRID: SCR_016387), or DNBSEQ-G400 (RRID: SCR_017980) platform.

Raw reads were assessed for quality using FastQC (v0.11.7, RRID: SCR_014583) and were trimmed for adapter clipping and removal of low-quality reads using Trim Galore (v.0.6.6, RRID: SCR_011847) with default settings. Trimmed reads were aligned to the hg38 reference human genome using HISAT2 software (v.2.1.0, RRID: SCR_015530; ref. 14) and then to the GRCh38.p13 reference human genome from the NCBI (RRID: SCR_006472) RefSeq database using the htseq-count tool in the Python package HTSeq (v.0.11.1, RRID: SCR_005514; ref. 15). Read counts were normalized to transcripts per million for downstream analysis.

Differential gene expression analysis was conducted using the R package DESeq2 (RRID: SCR_015687), applying a negative binomial model to raw count data (16).

For gene set enrichment analysis (GSEA), we utilized the GenePattern public server (https://cloud.genepattern.org/, RRID: SCR_003199) to perform single-sample GSEA (ssGSEA), which quantifies the enrichment levels of gene sets in individual samples with default parameters (17). This analysis included the CCC signature dataset (18) and MSigDB (RRID: SCR_016863) gene sets related to endoplasmic reticulum (ER) stress–associated pathways (GOBP_IRE1_mediated_unfolded_protein_response, GOBP_regulation_of_PERK_mediated_unfolded_protein_response, and GOBP_ATF6_mediated_unfolded_protein_response, H: hallmark gene sets). Principal component analysis (PCA; center = TRUE and scale = TRUE) was performed using data from ER stress–associated pathways on CCC organoids, serous organoids, normal organoids, multiple myeloma, TCGA ovarian cancer, and TCGA pancreatic adenocarcinoma datasets. The PCA analysis was conducted using the FactoMineR package (v2.9) and the factoextra package (v1.0.7) in R.

The data comparing the expression levels of *AGR2* knockout (KO) and control were analyzed using QIAGEN IPA (Qiagen, https://digitalinsights.qiagen.com/IPA, RRID: SCR_008653).

### Next-generation DNA sequencing

DNA was extracted from the organoid samples using the QIAamp DNA Mini Kit (Qiagen). Subsequently, sequencing libraries were constructed using the xGen Exome Research Panel (Integrated DNA Technologies) for DNA sequencing. The DNA was then subjected to WES on the NovaSeq 6000 platform with 150-bp paired-end reads.

Raw reads were assessed for quality with FastQC (v0.11.7, RRID: SCR_014583) and then aligned to the GRCh38 human reference genome with the Burrows–Wheeler aligner (v.0.7.17, RRID: SCR_010910) mem function (19). Duplicate reads were identified and marked using the Picard MarkDuplicates tool (v2.17.8, RRID: SCR_006525). The WES reads were filtered to retain only those aligned to the target region of the xGen Exome Research Panel. Quality scores were recalibrated using GATK4 (v.4.1.7, RRID: SCR_001876) BaseRecalibrator, resulting in the generation of the final binary alignment map files. Somatic single-nucleotide variants and short insertions and deletions were called with GATK Mutect2 and filtered with the normal panel. Mutations not labeled as “PASS” in GATK’s FilterMutectCalls were excluded. Additionally, mutations with a variant allele frequency of less than 0.05, a tumor sample read depth of less than 25, and a tumor sample ALT allele count of less than three were also discarded. Finally, an oncoplot displaying mutations in consensus genes from the Catalogue of Somatic Mutations in Cancer (Census_allMon Apr 5 13_35_42 2021.csv) was generated using the R package maftools (v.2.11.0, RRID: SCR_024519; ref. 20).

### AGR2 IHC

The tissue samples were fixed in 10% buffered formalin and embedded in paraffin following standard protocols. IHC was performed using an automated staining system (BOND-MAX) from Leica Biosystems. Unstained 4-μm sections of clinical specimens were deparaffinized, rehydrated, and incubated with a primary antibody against AGR2 (1:500 dilution, D9V2F, XP Rabbit mAb #13062, Cell Signaling Technology) at room temperature for 30 minutes. The primary antibody was then visualized using the BOND Polymer Refine Detection Kit (cat. #DS9800, Leica Biosystems) following the manufacturer’s instructions. Finally, the slides were counterstained with hematoxylin, dehydrated, and mounted. The staining was assessed based on the proportion of positive cells (0%–100%).

### Organoid and cell culture

The organoid culturing method used was previously reported (8). The organoid medium consisted of advanced DMEM/F12 (Thermo Fisher Scientific) supplemented with 2 mmol/L HEPES (Thermo Fisher Scientific), 1× GlutaMAX-I (Thermo Fisher Scientific), 1× B27 supplement (Thermo Fisher Scientific), 10 nmol/L Leu15-Gastrin I (Sigma-Aldrich), 1 mmol/L N-acetylcystein (Sigma-Aldrich), 100 ng/mL recombinant human insulin-like growth factor 1 (R&D Systems), 50 ng/mL recombinant human FGF-2 (Thermo Fisher Scientific), 20% Afamin/Wnt3a CM (JSR Life Sciences), 1 μg/mL human R-spondin (R&D Systems), 100 ng/mL Noggin (Thermo Fisher Scientific), 500 nmol/L A-83-01 (Tocris Bioscience), 200 U/mL penicillin/streptomycin (Thermo Fisher Scientific), and 10 μmol/L Y-27632 (Fujifilm Wako).

### Transmission electron microscopy

The detailed procedure was previously described (21). Briefly, the samples were pre-fixed with 2.5% glutaraldehyde in 0.1 mol/L phosphate buffer at 4°C for 16 hours and then fixed with 1% OsO_4_ in 0.1 mol/L phosphate buffer at 4°C for 2 hours. The specimens were dehydrated in a dilution series of 50% to 100% ethanol and in QY1 (n-butyl glycidyl ether) and embedded in a graded concentration of resin and QY1 and finally embedded in pure epoxy resin (100 g pure resin composed of 27.0 g methyl nadic anhydride, 51.3 g EPOK-812, 21.9 g dodecenylsuccinic anhydride, and 1.1 mL DMP-30, all from Okenshoji Co. Ltd.). The samples were then polymerized at 65°C for 72 hours. Ultrathin sections (80 nm) were prepared using an ultramicrotome (EM UC7, Leica Biosystems), collected on copper grids, and stained with uranyl acetate and lead citrate. The sections were examined under transmission electron microscopy (TEM; JEM-1400Plus, JEOL) at 100 kV. For quantitative evaluation of ER diameters, the length was measured using TEM center software in JEM-1400Plus at more than 50 independent locations in multiple fields of view.

### HTDS

A total of 2,000 cells/10 μL of Matrigel (Corning) mixed with 40 μL of culture medium were seeded on day 0 and cultured. An assay-ready plate (ARP) was prepared using EDR-384SX (BioTec). In the ARP, 1 μL of 1 mmol/L compound in DMSO or 3 μL of 1 mmol/L compound in water was dispensed in each well of a 384-well plate and frozen at −80°C. For the DMSO ARP, the thawed ARP was diluted with 99 μL of organoid culture medium per well, and 5 μL of the diluted solution was transferred to the organoid culture plate on day 3. For the water ARP, the thawed ARP was diluted with 27 μL of organoid culture medium per well, then further diluted to 10 μmol/L, and 5 μL of the diluted solution was transferred to the organoid culture plate on day 3. On day 6, 40 μL of CellTiter-Glo 3D (Promega) was added and analyzed with a GloMax plate reader (Promega). The inhibition efficiency (%) was calculated as [1 − (target well − average low control)/(average high control − average low control)] × 100. The survival rate (%) was defined as 100 − inhibition efficiency (%). The low control was 10 μmol/L doxorubicin, and the high control was DMSO.

### Animal study

NOD/Shi-scid IL-2Rγ knockout mice (6 weeks old, female) were obtained from In-Vivo Science. CCC organoids equivalent to 1 × 10^6^ cells suspended in Matrigel were subcutaneously transplanted. The extracted tumors were fixed in 10% buffered formalin, embedded in paraffin, and assessed after hematoxylin and eosin staining and IHC staining.

For drug-sensitivity assessment in xenografts, six patient-derived organoid xenograft (PDOX) mice were used for each drug. Drugs were administered 1 week after confirming tumor formation after organoid implantation. Bortezomib (1 mg/kg, twice a week), dinaciclib (25 mg/kg, three times a week), and DMSO (control, twice a week) were administered via the transperitoneal route. Tumor volume was calculated weekly using the formula [(major axis) × (minor axis)^2^]/2, and tumor weight was measured after excision. These experiments were performed with sextuplicate samples. All animal procedures were approved by the Keio University School of Medicine Animal Care Committee (approval no. A2022-046).

### Western blot analyses

Cultured organoids were collected using Cell Recovery Solution (Corning) and lysed with M-PER Mammalian Protein Extraction Reagent (Thermo Fisher Scientific) supplemented with Halt Protease and Phosphatase Inhibitor Cocktail, EDTA-free (Thermo Fisher Scientific). Protein concentration was quantified using the Bradford protein assay (Bio-Rad), and equal amounts of protein (10 μg) were loaded onto each lane of Mini-PROTEAN TGX Gels 4% to 20% (Bio-Rad). The proteins were then transferred to the membrane (Trans-Blot Turbo Transfer Pack, Bio-Rad), blocked, probed with the aforementioned antibodies using the iBind Automated Western System (Thermo Fisher Scientific), and visualized with the Amersham Imager 600 (GE Healthcare). The results were validated through two independent experiments.

### Cell growth assay

For the cell growth assay of CCC organoids, 1.5 × 10^3^ cells in 60 μL Matrigel with 1 mL culture medium were seeded and cultured. Cell viability was assessed using an ATP assay with CellTiter-Glo 3D Reagent (Promega) and GloMax (Promega) on days 3 and 7 (and day 14 for *AGR2*-KO 18-015). These experiments were performed in sextuplicate cultures, and the results were validated through three independent experiments.

### Cell cycle analysis

For the cell cycle analysis, 3 × 10^4^ cells in 60 μL Matrigel with 1 mL culture medium were seeded and cultured. Samples were dispersed using TrypLE Express (Thermo Fisher Scientific) and fixed with 70% ethanol at −20°C for 16 hours. After washing with PBS, the cells were resuspended in staining buffer (PBS with 100 μg/mL RNase A and 0.25 μg/mL 7-Amino-Actinomycin D; BD Biosciences). The CytoFLEX S flow cytometer (Beckman Coulter) was used for cell analysis, with data analyzed using CytoExpert software (Beckman Coulter). In this experiment, *AGR2*-KO organoids from clones B and C were used as the *AGR2*-KO groups, with triplicate samples included in each group.

### Drug-sensitivity testing

Drug-sensitivity testing was performed as previously described (8). Briefly, 1.5 × 10^3^ cells in 10 μL Matrigel with 40 μL culture medium were seeded and cultured. Drugs were administered 3 days after embedding, and cell viability was assessed 3 days later using CellTiter-Glo 3D Reagent (Promega) and GloMax (Promega). Drug concentrations ranged from 10 μmol/L to 100 pmol/L. Data analysis, including the calculation of IC_50_ values, was performed using GraphPad Prism 8 (GraphPad Software) and JMP version 17.0 (SAS Institute). Each experiment was conducted in triplicate (quintuplicate for assays with *AGR2*-KO organoids), and the results were validated through two independent experiments.

### Functional analysis after drug administration

Western blot analysis and TEM were conducted after drug administration. Initially, 1 × 10^4^ cells in 60 μL Matrigel with 1 mL culture medium were seeded and cultured. Subsequently, the cells were treated with bortezomib at concentrations of 10 and 100 nmol/L and dinaciclib at concentrations of 100 and 500 nmol/L. After 24 hours of drug administration, the cells or proteins were collected using the aforementioned method and used in the respective experiments.

### *AGR2* knockout by CRISPR/Cas9

We generated *AGR2*-KO CCC organoids (19-024 and 18-015) using the CRISPR/Cas9 system. The CRISPR knockout was performed as previously described (22) with minor modifications. Briefly, the CRISPR target sequence for single-guide RNA was designed as TCT​GGC​CAG​AGT​GTA​GGA​GA (forward primer: CAAAGACCACCTGTATTC and reverse primer: CTG​GGA​TAA​TAG​ACC​CTA​TAC). The single-guide RNA was co-electroporated with the GFP-puro piggyBAC transposon vector (PB513B-1, System Biosciences) to increase the knockout efficiency. After a 2-day hypothermic incubation at 30°C, organoids were treated with puromycin, and then puromycin-resistant clones were manually picked up and expanded. The knockout was confirmed by the biallelic introduction of frameshift mutations using the TIDE web tool.

### Statistical analysis

#### Gene expression analysis

The two-tailed Student/Welch unpaired *t* test and Mann–Whitney *U* test were used to analyze the differences between ssGSEA scores and sample characteristics of the two groups. A χ^2^ test was used to analyze the association between *AGR2* and *SLFN11* expressions. A heatmap was created using the R library ComplexHeatmap (RRID: SCR_017270), displaying the IC_50_ data of each compound for CCC organoids.

#### Transmission electron microscope

The diameters of the ER in the three groups were compared using the Dunnett test, with organoids without drug administration serving as the control.

#### Cell growth assay

The ATP concentrations on day 7 in the four groups were compared using the Dunnett test, with the *AGR2* wild-type organoid serving as the control.

#### Cell cycle analysis

The proportions of cells in the G_2_–M phase between the two groups were compared using the Wilcoxon test.

#### Survival analysis

Cumulative survival was estimated using the Kaplan–Meier method, and differences in progression-free survival were analyzed using the log-rank test.

#### Drug-sensitivity analysis

In the drug-sensitivity analysis using *AGR2*-KO organoids, the percentages of cell survival at 0.01 μmol/L for bortezomib and 10 μmol/L for carboplatin were compared using the Dunnett test, with the *AGR2* wild-type organoid serving as the control.

#### Animal study

The calculated tumor volume, tumor weight, and body weight of the mice in the two groups were compared using the Wilcoxon test.

All statistical and survival analyses mentioned above were conducted using JMP version 17.0 (SAS Institute), with the level of statistical significance set at a *P* value of less than 0.05.

### Study approval

Written informed consent was collected from all patients (Institutional Review Board number 20070081). All procedures involving mice were conducted in accordance with protocols approved by the Keio University School of Medicine. This study adhered to the Declaration of Helsinki and the Japanese Ethical Guidelines for Medical and Health Research Involving Human Subjects.

### Data availability

WES and RNA sequencing data are deposited in the Japanese Genotype-phenotype Archive under the accession number JGAS000764. Other data generated in this study are available upon request from the corresponding author.

## Results

### Establishment of a CCC biobank

**Figure 1:**
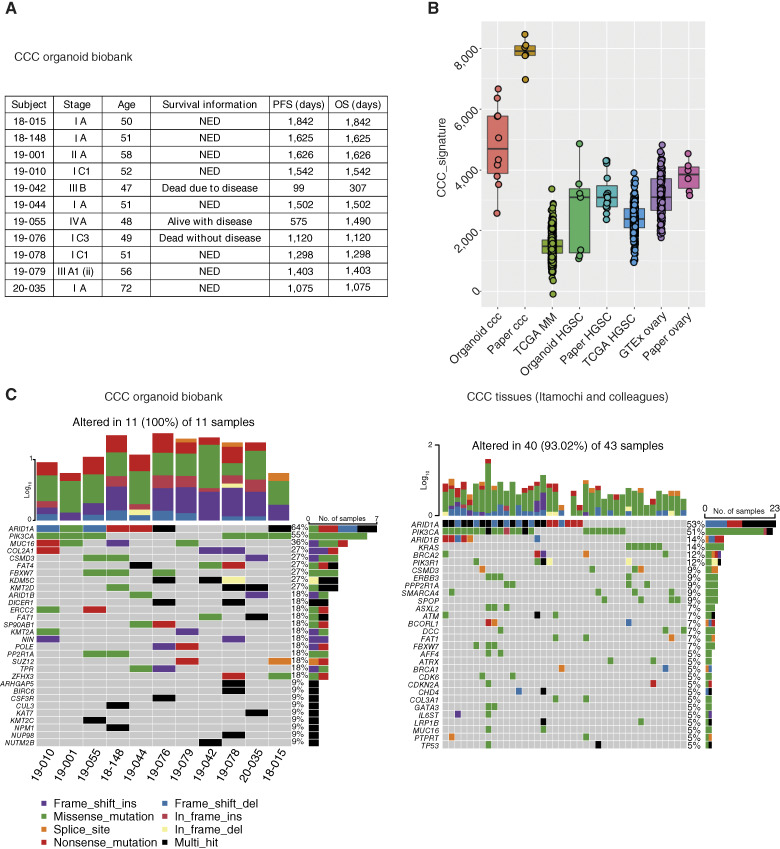
Establishment of a CCC organoid biobank. **A,** Clinicopathologic characteristics of the 11 cases of CCC from which organoids were derived. NED, no evidence of disease; OS, overall survival; PFS, progression-free survival. **B,** ssGSEA showing an activation of the CCC signature in CCC organoids. MM, multiple myeloma. **C,** Genomic characteristics of the 11 CCC organoids analyzed by exome sequencing (left) and 43 CCC tissues analyzed by WES [Itamochi and colleagues (23); right].

As of June 2022, a total of 57 ovarian cancer organoids (including borderline tumor) and 16 benign tissue organoids (14 normal fallopian tube organoids, one normal ovarian surface epithelium organoid, and one endometriotic cyst) were successfully established. Among them, 15 were derived from CCC, with 11 CCC organoids specifically used for this study (Fig. 1A). Transcriptome analysis of nine CCC organoids and seven HGSC organoids revealed that CCC organoids harbored a distinct CCC signature (18) compared with HGSC organoids or normal ovaries (Fig. 1B). Exome sequencing of the 11 CCC organoids showed that four organoids had both *PIK3CA* and *ARID1A* mutations, three had *ARID1A* mutations without *PIK3CA* mutations, and two did not have mutations in either *PIK3CA* or *ARID1A* [Fig. 1C (left)], reflecting the known mutational features of CCC [Fig. 1C (right); ref. 23]. From this, our established CCC organoid biobank is considered to reflect the characteristics of CCC in the real world, including the presence or absence of major genetic mutations. Notably, organoids derived from 19-042, a case with a poor prognosis, did not exhibit mutations in either *PIK3CA* or *ARID1A*.

### HTDS using CCC organoids

**Figure 2:**
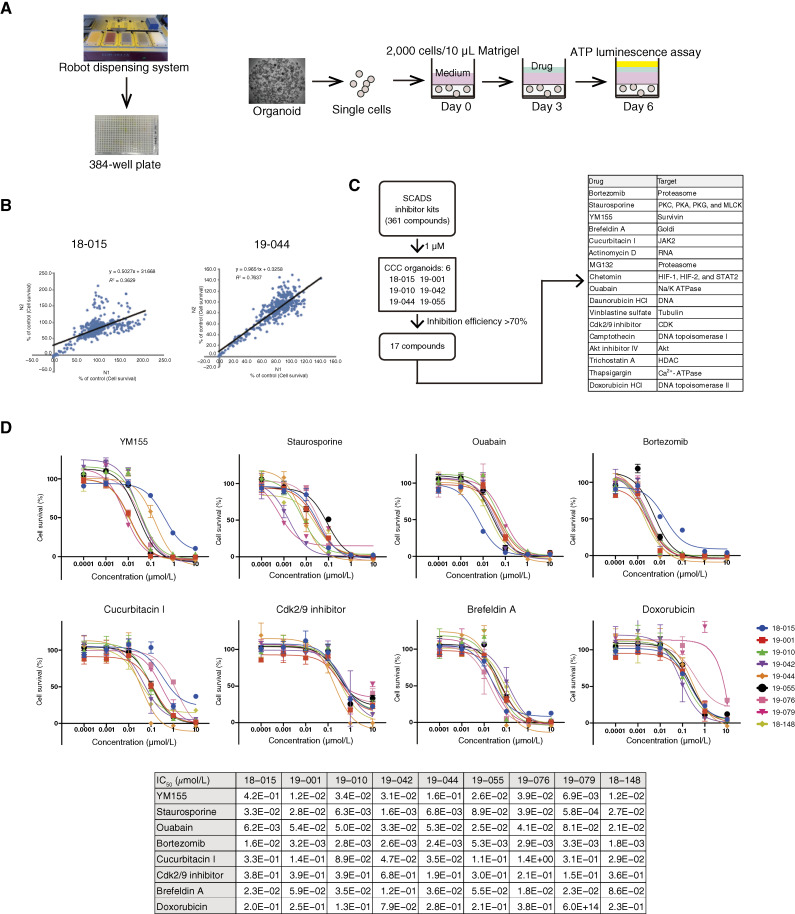
HTDS for CCC organoids using a 384-well plate. **A,** ATP luminescence-based HTDS system. **B,** Two independent HTDS showed reproducible results in two CCC organoids. **C,** Schematic presentation of HTDS with 361 compounds for six CCC organoids. HTDS identified 17 compounds with inhibition efficiencies over 70%. **D,** Dose–response curve of eight compounds for nine CCC organoids. The IC_50_ of each compound in each organoid is shown.

The organoid HTDS platform using a 384-well plate is depicted in Fig. 2A. The analysis of survival rates for 361 drugs was conducted twice, revealing concordance in two CCC organoids, 18-015 and 19-044 (Fig. 2B), thereby validating the reliability of the HTDS. Among the 361 compounds tested, 17 including YM155, staurosporine, ouabain, bortezomib (Velcade, PS-341), cucurbitacin I, Cdk2/9 inhibitor, brefeldin A, and doxorubicin were identified as candidate drugs, resulting in less than 30% survival of control in the six CCC organoids (Fig. 2C). Subsequently, drug-sensitivity testing was performed with the eight candidate compounds (YM155, staurosporine, ouabain, bortezomib, cucurbitacin I, Cdk2/9 inhibitor, brefeldin A, and doxorubicin) on nine CCC organoids. All compounds exhibited cytotoxic efficacy against CCC organoids at an IC_50_ level of less than 1 μmol/L (Fig. 2D). Notably, bortezomib demonstrated potent cytotoxicity (IC_50_ < 0.02 μmol/L) across all nine CCC organoids.

**Figure 3:**
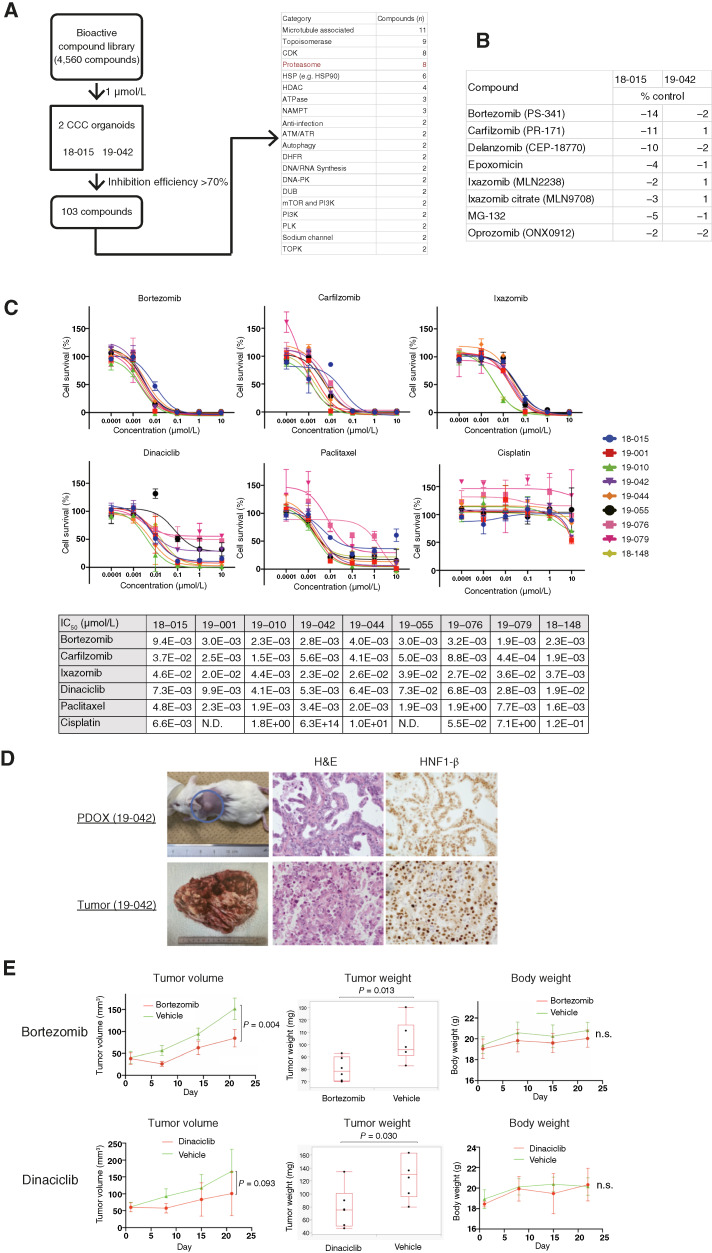
An HTDS with 4,560 compounds identified PIs and dinaciclib as candidate drugs for CCC, demonstrating effectiveness *in vivo*. **A,** Schematic presentation of HTDS with 4,560 compounds for two CCC organoids (18-015 and 19-042). DHFR, dihydrofolate reductase; HDAC, histone deacetylase. **B,** Results (% control) of eight PIs in the HTDS in two CCC organoids. **C,** Dose–response curve of three PIs (bortezomib, carfilzomib, and ixazomib), dinaciclib, paclitaxel, and cisplatin in nine CCC organoids (18-015, 19-001, 19-010, 19-042, 19-044, 19-055, 19-076, 19-079, and 18-148). The IC_50_ values of each compound in each organoid are shown. **D,** Histopathology of a PDOX (19-042) and its original tumor. H&E, hematoxylin and eosin. **E,***In vivo* experiments using PDOX treated with bortezomib or dinaciclib. Bortezomib treatment significantly reduced tumor volume (*P* = 0.004) and tumor weight (*P* = 0.013) without body weight loss. Dinaciclib treatment showed a tendency to reduce tumor volume (*P* = 0.093) and significantly reduce tumor weight (*P* = 0.030) without body weight loss. DUB, deubiquitinase; NAMPT, nicotinamide phosphoribosyltransferase; N.S., not significant; PLK, polo-like kinase; TOPK, T-LAK cell-originated protein kinase.

To further investigate effective drugs against CCC, HTDS using 4,560 compounds from the L1700 Bioactive Compound Library was conducted. HTDS on two CCC organoids (18-015 and 19-042) identified 103 drugs that were effective in both organoids, resulting in less than 30% survival compared with the control. The candidate drugs included 11 microtubule-associated drugs (such as cabazitaxel, colchicine, vinorelbine, and vinblastine), nine topoisomerase inhibitors (such as camptothecin, doxorubicin, epirubicin, and SN-38), eight proteasome inhibitors (PI), six HSP inhibitors, and four histone deacetylase inhibitors (dacinostat, panobinostat, quisinostat, and CUDC-907; Fig. 3A; Supplementary Table S1). Among the PIs tested, bortezomib, carfilzomib, delanzomib, epoxomicin, ixazomib, ixazomib citrate, MG-132, and oprozomib showed strong cytotoxicity compared with the control (Fig. 3B).

### PIs and dinaciclib demonstrated efficacy for CCC both *in vitro* and *in vivo*

PIs exhibited strong activity in screening tests, including a 361-drug screen and a 4,560-drug screen. Subsequently, we conducted drug sensitivity tests with FDA-approved multiple myeloma drugs, including bortezomib, carfilzomib, and ixazomib. Dinaciclib, identified as a candidate drug in the 4,560-drug screen, was also investigated because the Cdk (Cdk1, 2, 5, 9, and 12) inhibitor dinaciclib was reported as an encouraging single agent in multiple myeloma (24), and the Cdk2/9 inhibitor showed efficacy in CCC organoids (Fig. 2D). Bortezomib, carfilzomib, and ixazomib all demonstrated potent activity against nine CCC organoids (Fig. 3C). Dinaciclib also showed efficacy against CCC organoids, with an IC_50_ of less than 20 nmol/L. Paclitaxel exhibited efficacy in most CCC organoids, except for 19-076, whereas cisplatin was ineffective across all CCC organoids (Fig. 3C). Additionally, all six CCC organoids tested were resistant to carboplatin (Supplementary Fig. S1).

The PDOX mouse model using 19-042 exhibited pathologic features consistent with its original tumor and demonstrated hepatocyte nuclear factor 1 β positivity (Fig. 3D). Treatment with bortezomib in the PDOX model significantly reduced tumor growth without causing weight loss in the mice (*P* = 0.004 for tumor volume and *P* = 0.013 for tumor weight; Fig. 3E). Similarly, dinaciclib also exhibited a tumor-suppressing effect *in vivo* without affecting the weight of the mice (*P* = 0.093 for tumor volume and *P* = 0.030 for tumor weight; Fig. 3E). The effects of PIs and dinaciclib on CCC organoids were validated both *in vitro* and *in vivo*.

### Bortezomib induces unfolded protein response

PIs are drugs currently used for the treatment of multiple myeloma. One of the mechanisms of action of PIs is the accumulation of misfolded and unfolded proteins, resulting in excess ER stress and apoptosis via unfolded protein response (UPR; ref. 25). The effectiveness of PIs in multiple myeloma treatment is attributed to the elevated UPR levels in response to ER stress caused by immunoglobulin production (26). CCC cytoplasm contains not only glycogen but also many organelles, including the ER (27), and shares similarities with multiple myeloma in terms of a high possibility of arterial and venous thrombosis, known as Trousseau syndrome. To investigate the biological similarities between multiple myeloma and CCC, we examined the UPR pathway, which is initiated by three single-pass ER transmembrane proteins that possess an ER luminal domain capable of detecting misfolded proteins: IRE1α, PERK, and ATF6 (28).

**Figure 4:**
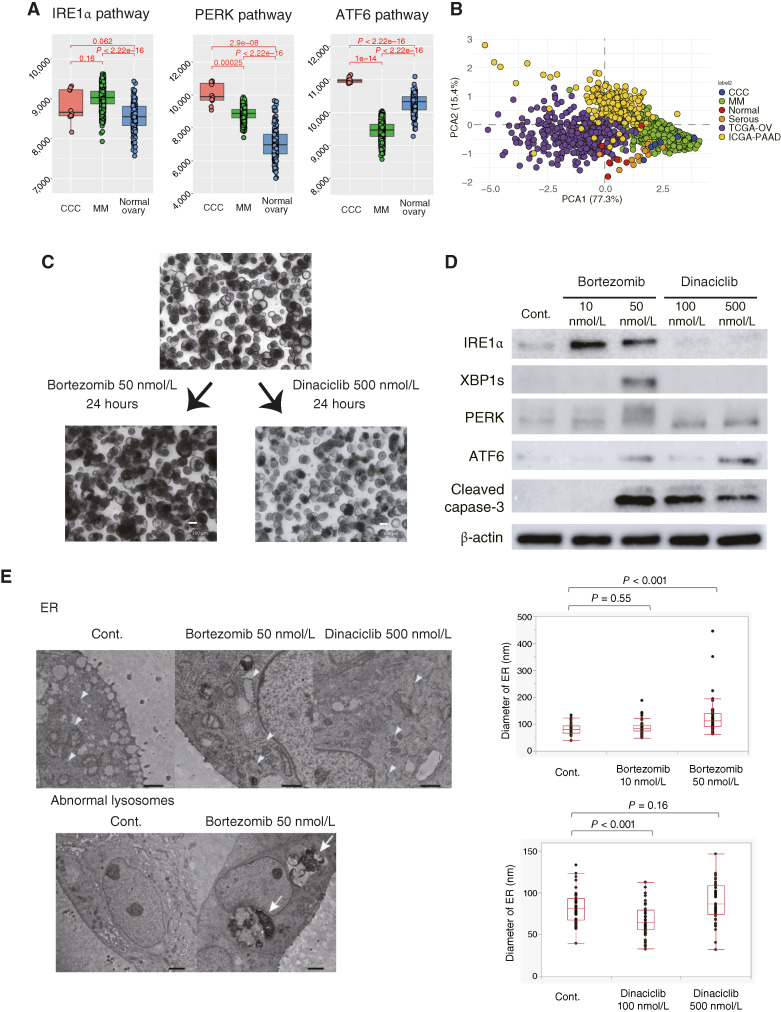
The activation of the UPR in CCC, with a higher level of activation observed following treatment with bortezomib. **A,** GSEA of the IRE1α pathway, PERK pathway, and ATF6 pathway in CCC, multiple myeloma from TCGA, and normal ovary from the Genotype-Tissue Expression database. **B,** PCA of ssGSEA using ATF6, IRE1α, and PERK pathways. TCGA-OV, TCGA ovarian cancer; TCGA-PAAD, TCGA pancreatic adenocarcinoma. **C,** Bright-field image of CCC organoid (19-042) treated with bortezomib (50 nmol/L, 24 hours) or dinaciclib (500 nmol/L, 24 hours). **D,** Immunoblot analysis of UPR markers (IRE1α, XBP1s, PERK, and ATF6) and an apoptosis marker (cleaved caspase-3) following treatment with bortezomib (10 or 50 nmol/L, 24 hours) or dinaciclib (100 or 500 nmol/L, 24 hours). **E,** TEM images after treatment with bortezomib (50 nmol/L, 24 hours) or dinaciclib (500 nmol/L, 24 hours). The arrowhead indicates the ER. Bortezomib treatment at 50 nmol/L significantly induced ER lumen dilatation (*P* < 0.001), whereas dinaciclib treatment did not. The arrow indicates the abnormal lysosome. Scale bars, 1 μm (top) and 2 μm (bottom). MM, multiple myeloma.

ssGSEA was conducted using the database of CCC, multiple myeloma, and normal ovaries, revealing that the IRE1α pathway and PERK pathway were upregulated in CCC and multiple myeloma compared with normal ovaries (IRE1α pathway, multiple myeloma: *P* < 2.22 × e^−16^ and CCC: *P* = 0.062 and PERK pathway, multiple myeloma: *P* < 2.22 × e^−16^ and CCC: *P* < 2.9 × e^−8^; Fig. 4A). Especially, the PERK pathway was highly activated in CCC compared with multiple myeloma. In CCC but not in multiple myeloma, the ATF6 pathway was significantly upregulated compared with the normal ovary (*P* < 2.22 × e^−16^). Furthermore, using the ssGSEA enrichment scores of the three UPR pathways, we performed PCA across multiple datasets, including CCC and multiple myeloma (Fig. 4B). The results demonstrated that CCC exhibited greater similarity to multiple myeloma than to HGSC or pancreatic cancer, both of which are also associated with Trousseau syndrome.

The treatment of the 19-042 CCC organoid with bortezomib resulted in a dark appearance under bright-field microscopic observation (Fig. 4C) and induced the expression of PERK, a key effector of cell death through ER stress, as well as IRE1α and XBP1s (Fig. 4D; ref. 29). In contrast, dinaciclib treatment did not lead to a dark appearance; it induced PERK and ATF6 but not IRE1α and XBP1s (Fig. 4D). TEM revealed significant dilatation of the ER lumen following bortezomib treatment, confirming the induction of ER stress (*P* = 0.55 for 10 nmol/L treatment and *P* < 0.001 for 50 nmol/L treatment; Fig. 4E). Conversely, dilatation of the ER lumen was not observed with dinaciclib treatment (*P* < 0.001 for 100 nmol/L treatment and *P* = 0.16 for 500 nmol/L treatment). Additionally, bortezomib treatment induced abnormal lysosomes (Fig. 4E). These findings suggest that bortezomib upregulates ER stress and the UPR, potentially contributing to the mechanism of action for CCC response to PIs.

### AGR2 as a therapeutic target in CCC

**Figure 5:**
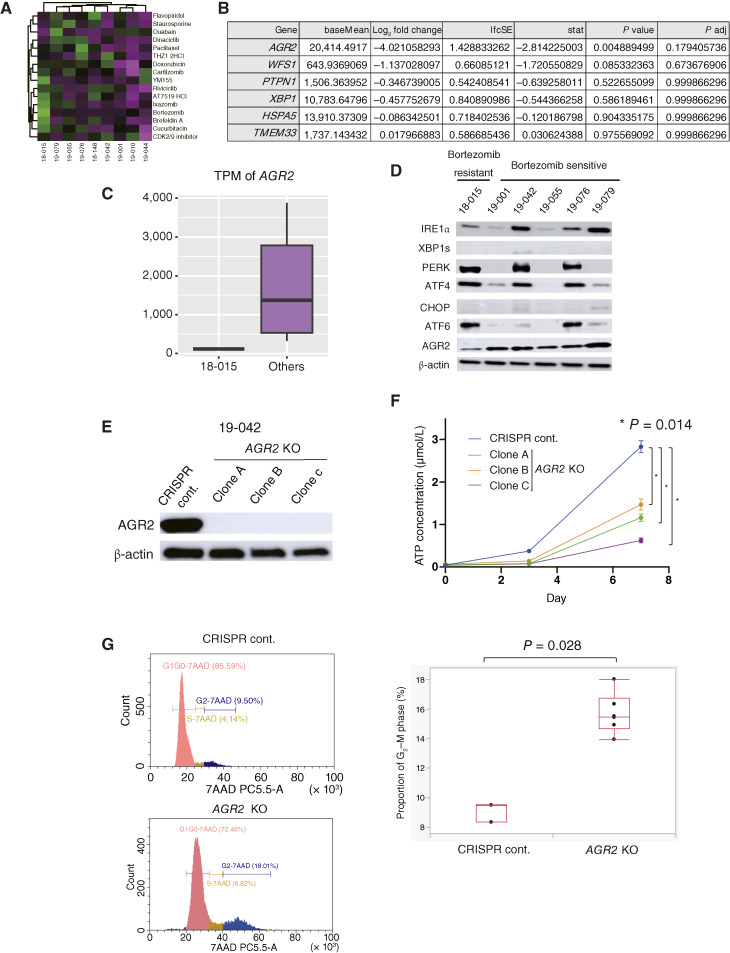
AGR2-high CCC organoids are sensitive to bortezomib, and *AGR2* knockout reduces tumor growth. **A,** Heatmap showing the IC_50_ of drugs selected through the HTDS of 361 compounds (Fig. 2) of nine organoids (18-015, 19-079, 19-055, 19-076, 18-148, 19-042, 19-001, 19-010, and 19-044). High, green; low, magenta. **B,** Genes shared in at least two of the UPR-related pathways (GOBP_IRE1_mediated_unfolded_protein_response, GOBP_regulation_of_PERK_mediated_unfolded_protein_response, and GOBP_ATF6_mediated_unfolded_protein_response). *AGR2* was significantly downregulated in the bortezomib-resistant 18-015 CCC organoid (*P* = 0.0049). **C,** Read count of *AGR2* in RNA sequencing of bortezomib-resistant (18-015) and other CCC organoids. **D,** Immunoblot analysis of UPR-related genes, including AGR2 in bortezomib-resistant (18-015) and other CCC organoids (19-001, 19-042, 19-055, 19-076, and 19-079). **E,** Immunoblot analysis of *AGR2*-KO CCC organoid (19-042) confirmed knockout of AGR2. **F,***AGR2*-KO organoids exhibited significantly slower growth than the control (*P* = 0.014). **G,** Cell cycle analysis of the *AGR2*-KO CCC organoid (19-042) showed a significant increase in the G_2_–M fraction compared with the control (*P* = 0.028).

We further investigated the mechanism underlying the sensitivity of CCC organoids to PIs. Drug candidates, including PIs, and their correlation with the IC_50_ for CCC organoids are depicted in Fig. 5A. Organoid 18-015 was identified as relatively resistant to PIs (Figs. 2D, 3C, and 5A). As IRE1α, PERK, and ATF6 pathways were upregulated in CCC (Fig. 4A), genes shared among these pathways were investigated. Among the gene sets related to these pathways, including GOBP_IRE1_mediated_unfolded_protein_response, GOBP_regulation_of_PERK_mediated_unfolded_protein_response, and GOBP_ATF6_mediated_unfolded_protein_response, *HSPA5, TMEM33*, *AGR2*, *PTPN11*, *XBP1*, and *WFS1* were found to be shared in at least two of the three pathways. Notably, *AGR2* expression varied significantly between bortezomib-sensitive and -resistant CCC organoids (*P* = 0.0049; Fig. 5B). Transcriptome analysis of UPR-related genes revealed that *AGR2* expression was upregulated in eight other CCC organoids compared with 18-015 (Fig. 5C). AGR2, a member of the protein disulfide isomerase family of ER-resident proteins, is induced by ER stress and plays a role in maintaining ER homeostasis (30). The protein level of AGR2 was found to be relatively low in organoid 18-015 compared with other CCC organoids (Fig. 5D). Interestingly, the expression levels of other proteins in the IRE1α, PERK, and ATF6 pathways were not associated with bortezomib sensitivity.

Next, we investigated the role of AGR2 in CCC organoids. *AGR2* CRISPR knockout resulted in significantly suppressed growth (Fig. 5E and F). The *AGR2*-KO organoids exhibited a significant increase in the G_2_–M population compared with the control (*P* = 0.028; Fig. 5G). However, in the AGR2-low CCC organoid (18-015), *AGR2* knockout did not affect cell growth (Supplementary Fig. S2). This suggests that there is an AGR2 dependency in AGR2-high CCC but not in AGR2-low CCC.

**Figure 6:**
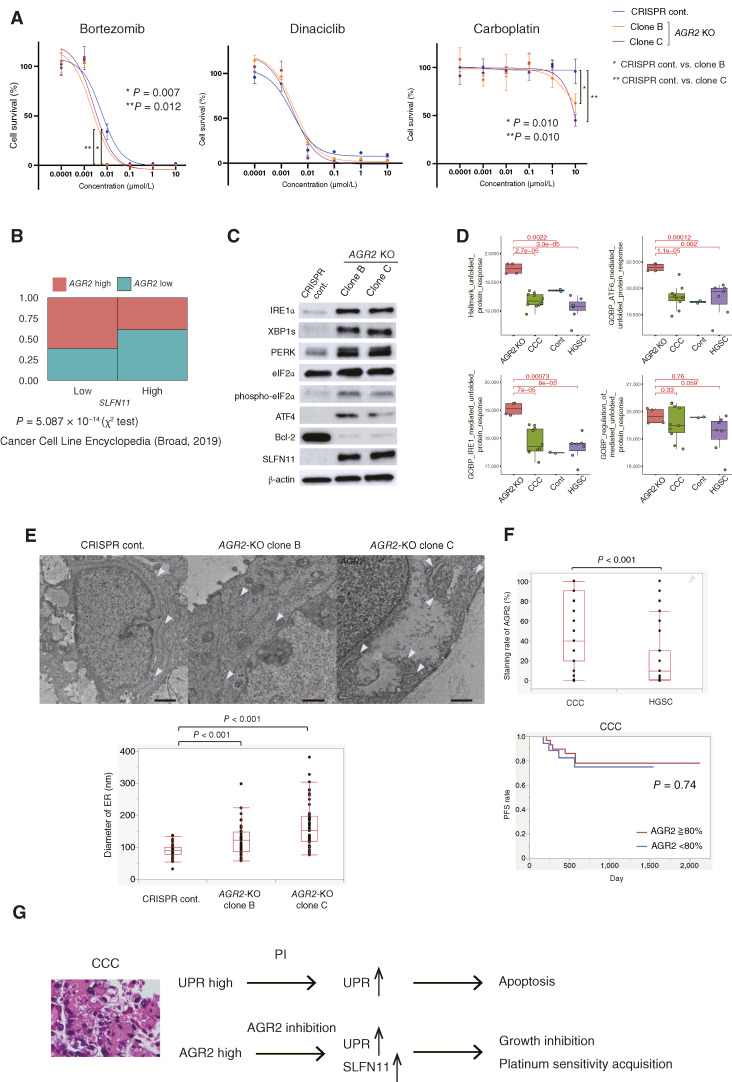
*AGR2* knockout sensitizes CCC organoids to carboplatin through upregulation of SLFN11 and induces UPR and ER stress. **A,** Dose–response curve of bortezomib, dinaciclib, and carboplatin in *AGR2*-KO organoids. *AGR2*-KO organoids showed increased sensitivity to bortezomib (*P* = 0.007 and 0.012) and carboplatin (both *P* = 0.010) but not to dinaciclib. **B,** Correlation between *AGR2* and *SLFN11* mRNA in the Cancer Cell Line Encyclopedia dataset (Broad, 2019; ref. 52). *AGR2* expression was significantly related to low *SLFN11* expression (*P* = 5.087 × 10^−14^). **C,** Immunoblot analysis of UPR pathway genes and SLFN11 in *AGR2*-KO CCC organoids. **D,** Activation of the UPR pathway in *AGR2*-KO CCC organoids (Hallmark_unfolded_protein_response, *P* = 0.0022; GOBP_ATF6_mediated_ unfolded_protein_response, *P* = 0.00012; GOBP_IRE1_mediated_unfolded_protein_response, *P* = 0.00073; and GOBP_regulation_of_PERK_mediated_unfolded_protein_response, *P* = 0.76, compared with the control). **E,** TEM images the of *AGR2*-KO CCC organoid. The arrowhead indicates the ER. *AGR2* knockout showed significant ER lumen dilatation (clone B, *P* < 0.001 and clone C, *P* < 0.001). **F,** Higher AGR2 expression in CCC (*n* = 51) than in HGSC (*n* = 43; *P* < 0.001). AGR2 expression was not correlated with progression-free survival in CCC (*P* = 0.74). **G,** Schematic representation of the identified mechanisms in this study. CCC exhibits high UPR, which is further upregulated by PIs to induce apoptosis. There are many AGR2-high CCCs, and AGR2 inhibition upregulates UPR and SLFN11 expression, leading to growth inhibition and platinum sensitivity.

Next, the effect of *AGR2* knockout on sensitivity to PI was investigated. *AGR2*-KO CCC organoids showed more sensitivity to bortezomib (*P* = 0.007 and 0.013 for different clones; Fig. 6A). The median cell survival percentage in *AGR2*-KO organoids (clone B and clone C) at 0.01 μmol/L bortezomib was 0.1% and 0.9%, respectively, whereas in the control organoid, it was 36.4%. As shown in Fig. 3C and Supplementary Fig. S1, CCC organoids were all resistant to cisplatin or carboplatin. However, *AGR2*-KO CCC organoids exhibited sensitivity to carboplatin (*P* = 0.010 and 0.010 for different clones; Fig. 6A). At 10 μmol/L carboplatin, *AGR2*-KO organoids (clone B and clone C) exhibited median cell survival rates of 64.2% and 42.8%, respectively, whereas the control organoid maintained a substantially higher viability at 96.3%. These findings suggest that AGR2, which is upregulated in CCC, could serve as a potential therapeutic target.

To further investigate the mechanism underlying the increased sensitivity of *AGR2*-KO organoids to both bortezomib and carboplatin, transcriptome analysis was conducted. The *AGR2*-KO CCC organoid exhibited elevated expression of *SLFN11* (Supplementary Table S2). SLFN11 is a putative DNA/RNA helicase that enhances the sensitivity of cancer cells to DNA-damaging agents, such as topoisomerase I/II inhibitors, alkylating agents like carboplatin and cisplatin, and DNA synthesis inhibitors such as gemcitabine (31). Indeed, *AGR2* expression showed a negative correlation with *SLFN11* expression in the Cancer Cell Line Encyclopedia dataset (Fig. 6B). The upregulation of SLFN11 protein levels in *AGR2*-KO organoids was confirmed (Fig. 6C). Additionally, *AGR2*-KO organoids exhibited increased expression of IRE1α, XBP1s, PERK, phospho-eIF2α, and ATF4, along with reduced expression of the anti-apoptotic protein, Bcl-2 (Fig. 6C). GSEA revealed significant upregulation of the UPR, particularly the ATF6 and IRE1α pathways, in *AGR2*-KO organoids compared with the control (UPR, *P* = 0.0022; ATF6, *P* = 0.00012; and IRE1α, *P* = 0.00073; Fig. 6D), confirming increased ER stress and UPR in *AGR2*-KO cells. Ingenuity pathway analysis further confirmed activation of the UPR pathway in *AGR2*-KO CCC organoids (Supplementary Fig. S3). In addition, *AGR2*-KO organoids exhibited ER dilatation compared with the control (*P* < 0.001 and *P* < 0.001 for different clones), indicating that *AGR2*-KO induces ER stress (Fig. 6E). This suggests that the enhancement of *AGR2*-KO–induced ER stress may contribute to the increased sensitivity to bortezomib. *AGR2* knockout also suppressed Wnt–β-catenin signaling (*P* = 0.042), the p53 pathway (*P* = 0.0032), epithelial–mesenchymal transition (*P* = 0.0057), notch signaling (*P* = 0.024), coagulation (*P* = 5.5 × e^−5^), and hypoxia (*P* = 0.0039), which are upregulated in CCC compared with HGSC, suggesting that AGR2 suppression is a promising therapeutic strategy for CCC (Supplementary Fig. S4).

*AGR2* knockout in the 18-015 organoid (with low AGR2 expression and modest bortezomib resistance) resulted in AGR2 loss without affecting cell proliferation, bortezomib sensitivity, or SLFN11 levels (Supplementary Figs. S2 and S5). These results suggest that AGR2 does not play a significant role in CCC, with low AGR2 expression observed. IHC analysis revealed a significant increase in AGR2 expression in CCC compared with HGSC (*P* < 0.001). Additionally, AGR2 had no significant effect on progression-free survival in CCC (*P* = 0.74; Fig. 6F). From these, it was suggested that although AGR2 expression is typically high in CCC, a subset of CCC with low AGR2 expression possesses distinct biological characteristics compared with AGR2-high CCC.

## Discussion

Advanced or recurrent CCC is a devastating disease that often does not respond well to conventional platinum-based chemotherapy. Although the proportion of CCC among ovarian cancers is low in Western countries, it is relatively high in Japan. The reason for this disparity is not fully understood but may be attributed to racial differences or environmental factors. Among cases of CCC, the *ARID1A* mutation is present in 41.5% and loss of ARID1A expression is observed in 75.6%, suggesting a crucial role of ARID1A in CCC biogenesis (4). Research has explored drugs targeting *ARID1A* loss or mutation, including those affecting glutathione metabolism (32), EZH2 (33), histone deacetylase (34), glutaminase (35), and the mevalonate pathway (36), which have shown promising results. Additionally, *PIK3CA* mutations are common, occurring in 30% of CCC cases (3), and therapies targeting the phosphatidylinositol-3-kinase pathway have been investigated. Our findings revealed the effectiveness of PIs in CCC organoids, regardless of *ARID1A* or *PIK3CA* mutations.

The dipeptide boronic acid analogue bortezomib is a potent, highly selective, and reversible inhibitor of the 26S proteasome complex (26). The ER is responsible for the biosynthesis of secreted and transmembrane proteins, in which proteins undergo folding, glycosylation, and lipidation processes. Conditions such as a high load of secreted proteins, the presence of misfolded proteins, and impaired glycosylation, collectively known as “ER stress,” lead to the accumulation of misfolded or unfolded proteins in the ER. In response to ER stress, cells trigger a series of signaling pathways, known as UPRs, initially aiming to restore cellular homeostasis but can trigger apoptosis if the stress persists (37). The initial phase of the UPR involves promoting the removal of unfolded proteins from the ER through a process called ER-associated degradation. PIs inhibit ER-associated degradation, leading to the accumulation of unfolded proteins and exacerbating ER stress. Treatment with bortezomib activates PERK and eIF2α phosphorylation in multiple myeloma cells, followed by the induction of ATF4 and CHOP (38). In CCC organoids, bortezomib also activates IRE1α, XBP1s, PERK, and ATF4, ultimately triggering an apoptotic pathway.

PIs are used as therapeutics for multiple myeloma, a malignant tumor originating from post-germinal center B lymphocytes. Multiple myeloma is characterized by the excessive production and secretion of immunoglobulins, leading to ER stress and disruption of proteostasis in the ER. Multiple myeloma cells rely on the UPR for survival (37) as it is highly active in multiple myeloma cells and becomes more pronounced as the disease progresses (39). Although the UPR promotes multiple myeloma cell survival and disease progression, exposure to exogenous ER stress can shift the balance between pro-survival and pro-apoptotic signals toward cell death. Our analysis indicates that the UPR is also highly activated in CCC and multiple myeloma, even compared with HGSC or pancreatic adenocarcinoma (Fig. 4A and B). In addition, thromboembolism is common in patients with CCC, partly attributed to elevated tissue factor levels and the production of tissue factor–rich extracellular vesicles by CCC cells (40). The abundant cytoplasm of CCCs contains many intracellular organelles, possibly reflecting their abundant protein production capacity. The shared characteristics between CCC and multiple myeloma suggest a potential therapeutic benefit of PIs in CCC treatment.

Bortezomib has been reported to activate the cGAS/STING pathway and induce type 1 IFN production in multiple myeloma (41). Immune checkpoint inhibitors have demonstrated effectiveness in some cases of CCC (42), suggesting that a combination of PIs and immune checkpoint inhibitors may be a promising therapeutic approach for CCC.

Our HTDS revealed that both PIs and dinaciclib are effective against CCC *in vitro* and *in vivo.* Dinaciclib, a pan-Cdk inhibitor, has shown effectiveness against platinum-sensitive and -resistant ovarian cancer cells (43), and it has synergistic effects with cisplatin in ovarian cancer (44). A recent study demonstrated the efficacy of dinaciclib in dramatically reducing metastatic burden in a patient-derived xenograft cell line orthotopic model of a patient with osteosarcoma (45). The Cdk1/2/5/9 expression, especially Cdk1, was found to be higher in dinaciclib-sensitive CCC organoids (19-055, 19-076, and 19-079: resistant vs. the other six sensitive CCC organoids; Supplementary Table S3). Although not statistically significant, Cdk2/5/9 also showed log fold change values lower than 0, indicating a trend toward higher expression in the sensitive organoids, further supporting the idea that CCCs with high expression of these Cdks may benefit from dinaciclib treatment.

We identified *AGR2* as a gene associated with the UPR in CCC. AGR2 is a resident ER protein that is highly expressed in mucus-secreting cells and endocrine tissues (46). AGR2 has been shown to promote cancer cell proliferation, invasion, and resistance to chemotherapy, and it may play a crucial role in regulating the ER’s ability to adapt to physiologic stress (47). In esophageal Barrett epithelium, AGR2 acts as a p53 inhibitor by inhibiting p53 phosphorylation at Ser15 and Ser392 (48). In pancreatic carcinogenesis, chronic ER stress leads to an inflammatory state that, along with *KRAS* mutations, induces AGR2 expression and contributes to pancreatic cancer development (49). Our study revealed significantly higher AGR2 expression in CCC than in HGSC (Fig. 6F), with AGR2 playing a crucial role in AGR2-high CCC but having minimal effect in AGR2-low CCC (Supplementary Figs. S2 and S5). In AGR2-high CCC, *AGR2* knockout induced ER stress, highlighting AGR2’s essential role in UPR homeostasis. Initially, we predicted that bortezomib sensitivity would decrease with *AGR2* knockout as CCC organoids with high AGR2 expression showed high sensitivity to bortezomib. However, contrary to our expectation, *AGR2* knockout further enhanced sensitivity to bortezomib. This observation suggests that organoids that originally had high AGR2 expression to cope with severe ER stress became exposed to even greater ER stress because of *AGR2* knockout, making them more susceptible to ER stress–mediated cell death induced by bortezomib. This hypothesis is supported by the finding that AGR2 contributes to the maintenance of ER homeostasis, as well as by our results showing that ER stress was enhanced in *AGR2*-KO organoids.

Interestingly, AGR2-deficient CCC organoids showed increased vulnerability to platinum drugs, which may be because of SLFN11 upregulation and Bcl-2 downregulation in CCC organoids. Previous studies have shown that SLFN11-positive CCCs exhibit heightened sensitivity to platinum drugs (50). The mechanism by which AGR2 regulates SLFN11 remains unclear, but SLFN11 has been reported to protect cells from UPR-induced cell ubiquitination caused by misfolded proteins (51). Therefore, the upregulation of SLFN11 in response to *AGR2* knockout may be a consequence of UPR activation. These findings suggest that AGR2 could serve as a potential therapeutic target for cancers with high AGR2 expression.

This study has several limitations. Among the 11 ovarian CCC organoids used, only three cases were advanced stage (stage III or higher), which may not constitute a sufficient CCC organoid to evaluate platinum sensitivity. Additionally, although dinaciclib was also identified in the drug screening, it did not induce ER stress as shown in Fig. 4C and D. The mechanism by which dinaciclib effectively exerts cytotoxic effects on CCC requires further investigation.

In conclusion, UPR is elevated in CCC, rendering it susceptible to PIs and dinaciclib, as demonstrated by HTDS using a CCC organoid biobank. AGR2 is highly activated in CCC, and inhibiting AGR2 leads to increased UPR, shifting the balance toward apoptosis and acquiring platinum sensitivity, possibly due to SLFN11 upregulation (Fig. 6G).

## Supplementary Material

Supplementary Figure S1. Dose–response curve of carboplatin in six clear cell ovarian cancer organoids CCC, clear cell ovarian cancer

Supplementary Figure S2. AGR2-knockout (KO) in AGR2-low clear cell ovarian cancer (CCC) did not suppress cell growth A, Immunoblot analysis confirmed AGR2 KO. B, AGR2 KO did not suppress cell growth in AGR2-low CCC organoid (18-015).

Supplementary Figure S3 QIAGEN Ingenuity Pathway Analysis results for unfolded protein response, filtered by DESeq2 analysis of AGR2 KO vs. control with abs(logFC) of >1.5 (p < 0.0005) (QIAGEN Inc., https://digitalinsights.qiagen.com/IPA). In the molecule, pink indicates actual upregulation, green indicates actual downregulation, orange indicates predicted upregulation, and blue indicates predicted downregulation.

Supplementary Figure S4 Box plot showing Single-sample Gene Set Enrichment Analysis (ssGSEA) scores significantly different between AGR2-knockout organoids and controls (HALLMARK_WNT_BETA_CATENIN_SIGNALING, HALLMARK_P53_PATHWAY, HALLMARK_PEROXISOME, HALLMARK_EPITHELIAL_MESENYCHYMAL_TRANSITION, HALLMARK_NOTCH_SIGNALING, HALLMARK_COAGULATION, HALLMARK_PROTEIN_SECRETION, HALLMARK_HYPOXIA). CCC, clear cell ovarian cancer; cont., control; HGSC, high-grade serous ovarian cancer

Supplementary Figure S5. Immunoblot analysis of AGR2-knockout 18-015 organoid showing no upregulation of SLFN11

Supplementary Table S1 Compounds with inhibition efficiency over 70% (n = 103) in two clear cell ovarian cancer organoids (18-015, 19-042).

Supplementary Table S2. Gene expression differences between AGR2-knockout clear cell ovarian cancer organoids and controls (19-042)

Supplementary Table S3 Cdk1/2/5/9 gene expression differences between dinaciclib-resistant CCC organoids (19-055, 19-076, and 19-079) vs. the dinaciclib-sensitive CCC organoids (180-015, 19-001, 19-010, 19-042, 19-044, 18-148). A log fold change (logFC) greater than 0 was considered to indicate higher gene expression in dinaciclib-resistant samples.
