# Supplementary material for: High-Throughput Drug Screening of Clear Cell Ovarian Cancer Organoids Reveals Vulnerability to Proteasome Inhibitors and Dinaciclib and Identifies AGR2 as a Therapeutic Target
Source: Cancer Res Commun. 2025 Jun 25;5(6):1018–33. doi: 10.1158/2767-9764.CRC-25-0024 (PMC12188421; doi:10.1158/2767-9764.CRC-25-0024)
Supplement: Supplementary Figure S1. — Dose–response curve of carboplatin in six clear cell ovarian cancer organoids CCC, clear cell ovarian cancer [file crc-25-0024_supplementary_figure_s1.suppsf1.pdf]

Yoshimura T et al. Supplementaty Figure S1

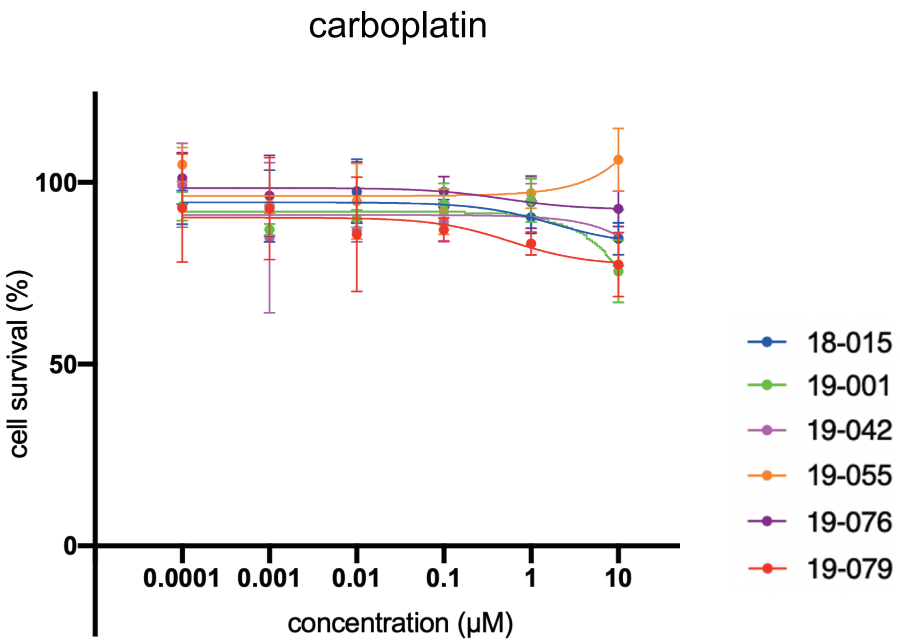

Supplementary Figure S1. Dose-response curve of carboplatin in six clear cell ovarian cancer organoids

CCC, clear cell ovarian cancer
