# Supplementary material for: High-Throughput Drug Screening of Clear Cell Ovarian Cancer Organoids Reveals Vulnerability to Proteasome Inhibitors and Dinaciclib and Identifies AGR2 as a Therapeutic Target
Source: Cancer Res Commun. 2025 Jun 25;5(6):1018–33. doi: 10.1158/2767-9764.CRC-25-0024 (PMC12188421; doi:10.1158/2767-9764.CRC-25-0024)
Supplement: Supplementary Figure S2. — AGR2-knockout (KO) in AGR2-low clear cell ovarian cancer (CCC) did not suppress cell growth A, Immunoblot analysis confirmed AGR2 KO. B, AGR2 KO did not suppress cell growth in AGR2-low CCC organoid (18-015). [file crc-25-0024_supplementary_figure_s2.suppsf2.pdf]

Yoshimura T et al. Supplementaty Figure S2

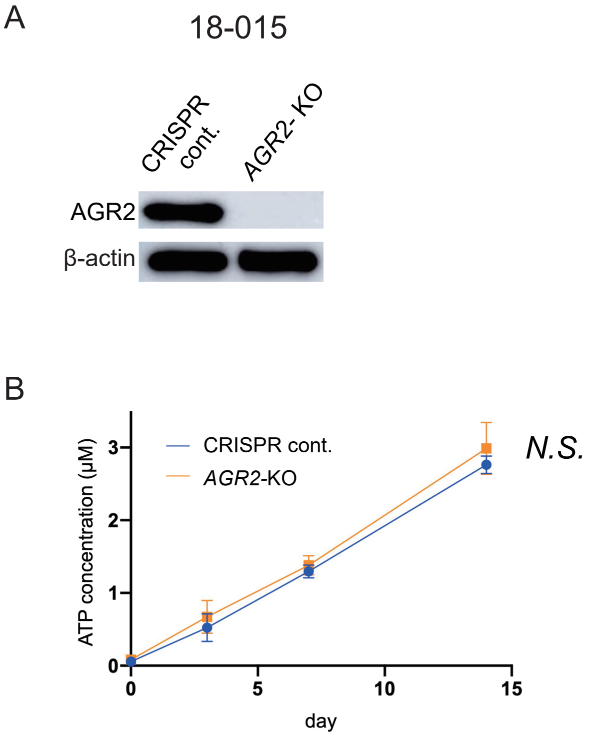

Supplementary Figure S2. *AGR2*-knockout (KO) in AGR2-low clear cell ovarian cancer (CCC) did not suppress cell growth

A, Immunoblot analysis confirmed AGR2 KO. **B**, *AGR2* KO did not suppress cell growth in AGR2-low CCC organoid (18-015).
