# Supplementary material for: High-Throughput Drug Screening of Clear Cell Ovarian Cancer Organoids Reveals Vulnerability to Proteasome Inhibitors and Dinaciclib and Identifies AGR2 as a Therapeutic Target
Source: Cancer Res Commun. 2025 Jun 25;5(6):1018–33. doi: 10.1158/2767-9764.CRC-25-0024 (PMC12188421; doi:10.1158/2767-9764.CRC-25-0024)
Supplement: Supplementary Figure S3 — QIAGEN Ingenuity Pathway Analysis results for unfolded protein response, filtered by DESeq2 analysis of AGR2 KO vs. control with abs(logFC) of >1.5 (p < 0.0005) (QIAGEN Inc., https://digitalinsights.qiagen.com/IPA). In the molecule, pink indicates actual upregulation, green indicates actual downregulation, orange indicates predicted upregulation, and blue indicates predicted downregulation. [file crc-25-0024_supplementary_figure_s3_suppsf3.pdf]

# Yoshimura T et al. Supplementaty Figure S3

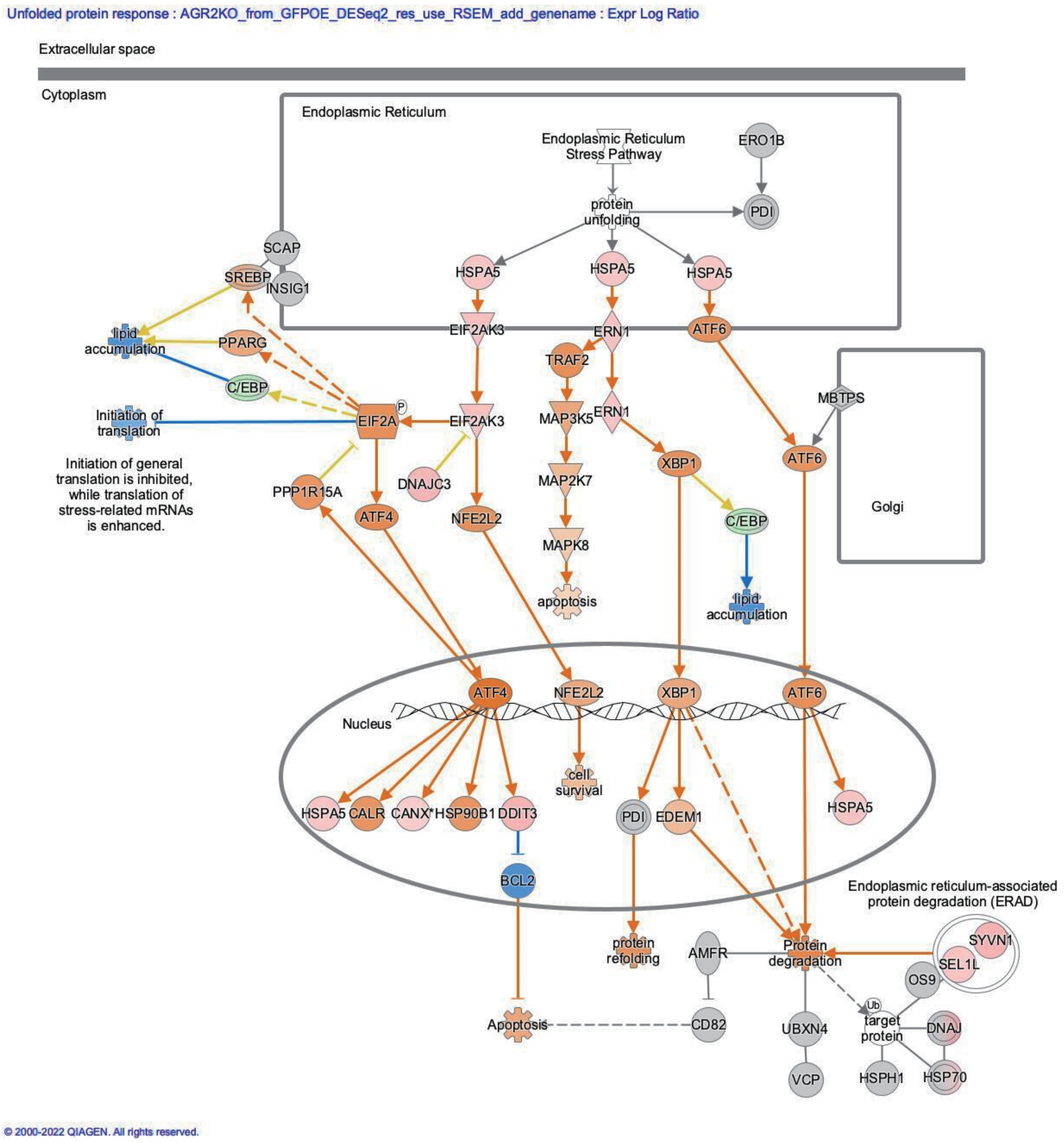


© 2000-2022 QIAGEN. All rights reserved.

**Supplementary Figure S3.** QIAGEN Ingenuity Pathway Analysis results for unfolded protein response, filtered by DESeq2 analysis of *AGR2* KO *vs*. control with abs(logFC) over 1.5 (*p* <0.0005) (QIAGEN Inc., https://digitalinsights.qiagen.com/IPA). In the molecule, pink indicates actual upregulation, green indicates actual downregulation, orange indicates predicted upregulation, and blue indicates predicted downregulation.
