# Supplementary material for: High-Throughput Drug Screening of Clear Cell Ovarian Cancer Organoids Reveals Vulnerability to Proteasome Inhibitors and Dinaciclib and Identifies AGR2 as a Therapeutic Target
Source: Cancer Res Commun. 2025 Jun 25;5(6):1018–33. doi: 10.1158/2767-9764.CRC-25-0024 (PMC12188421; doi:10.1158/2767-9764.CRC-25-0024)
Supplement: Supplementary Figure S4 — Box plot showing Single-sample Gene Set Enrichment Analysis (ssGSEA) scores significantly different between AGR2-knockout organoids and controls (HALLMARK_WNT_BETA_CATENIN_SIGNALING, HALLMARK_P53_PATHWAY, HALLMARK_PEROXISOME, HALLMARK_EPITHELIAL_MESENYCHYMAL_TRANSITION, HALLMARK_NOTCH_SIGNALING, HALLMARK_COAGULATION, HALLMARK_PROTEIN_SECRETION, HALLMARK_HYPOXIA). CCC, clear cell ovarian cancer; cont., control; HGSC, high-grade serous ovarian cancer [file crc-25-0024_supplementary_figure_s4_suppsf4.pdf]

Yoshimura T et al. Supplementaty Figure S4

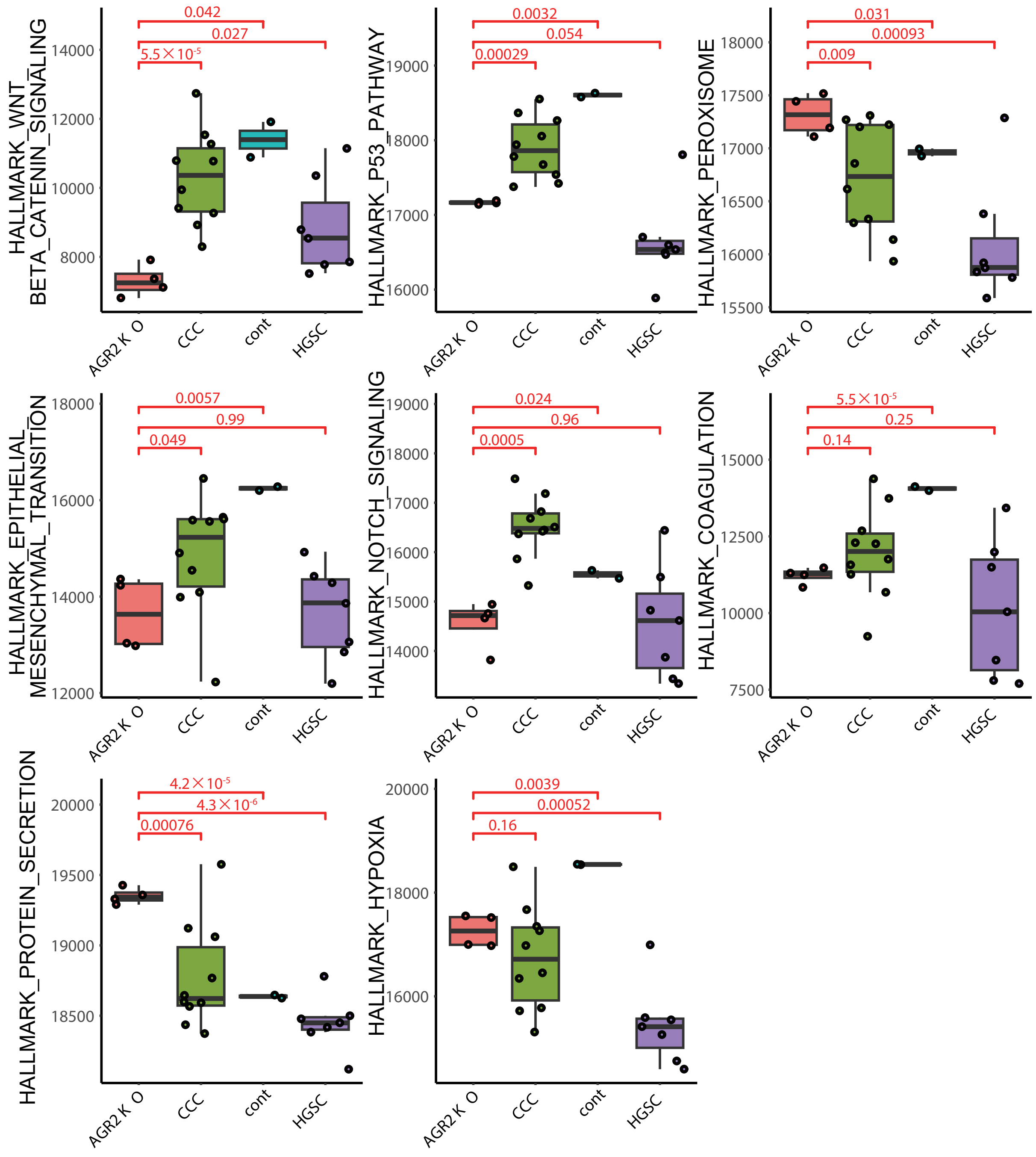

Supplementary Figure S4. Box plot showing Single-sample Gene Set Enrichment Analysis (ssGSEA) scores significantly different between *AGR2*-knockout organoids and controls (HALLMARK_WNT_BETA_CATENIN_SIGNALING, HALLMARK_P53_PATHWAY, HALLMARK_PEROXISOME, HALLMARK_EPITHELIAL_MESENYCHYMAL_TRANSITION HALLMARK_NOTCH_SIGNALING, HALLMARK_COAGULATION, HALLMARK_PROTEIN_SECRETION, HALLMARK_HYPOXIA). CCC, clear cell ovarian cancer; cont., control; HGSC, high-grade serous ovarian cancer
