# Supplementary material for: High-Throughput Drug Screening of Clear Cell Ovarian Cancer Organoids Reveals Vulnerability to Proteasome Inhibitors and Dinaciclib and Identifies AGR2 as a Therapeutic Target
Source: Cancer Res Commun. 2025 Jun 25;5(6):1018–33. doi: 10.1158/2767-9764.CRC-25-0024 (PMC12188421; doi:10.1158/2767-9764.CRC-25-0024)
Supplement: Supplementary Figure S5. — Immunoblot analysis of AGR2-knockout 18-015 organoid showing no upregulation of SLFN11 [file crc-25-0024_supplementary_figure_s5.suppsf5.pdf]

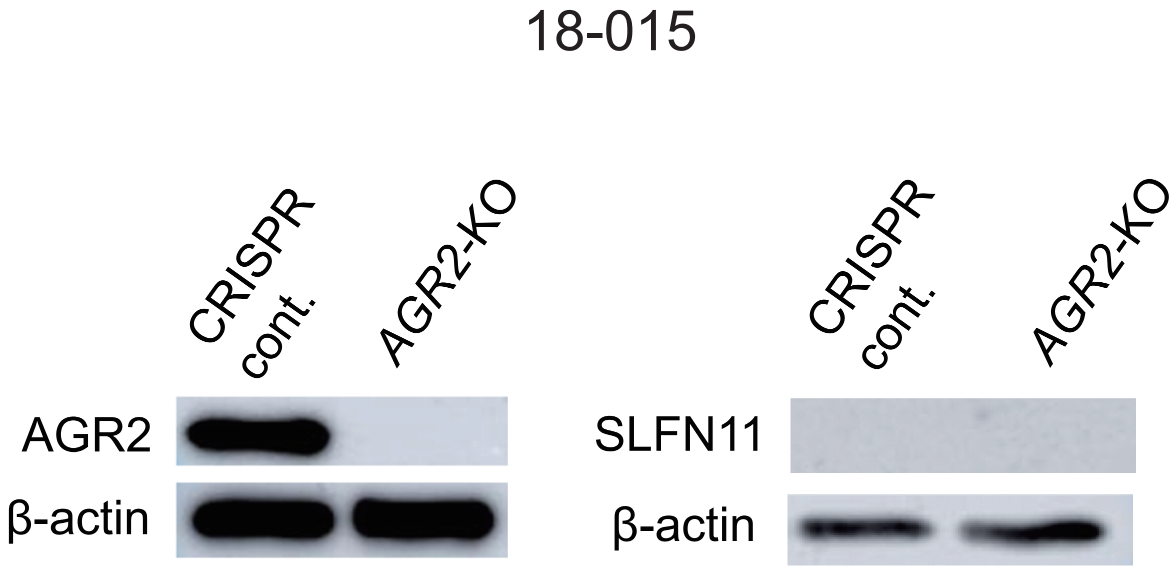

Supplementary Figure S5. Immunoblot analysis of *AGR2*-knockout 18-015 organoid showing no upregulation of *SLFN11*
