# Supplementary material for: High-Throughput Drug Screening of Clear Cell Ovarian Cancer Organoids Reveals Vulnerability to Proteasome Inhibitors and Dinaciclib and Identifies AGR2 as a Therapeutic Target
Source: Cancer Res Commun. 2025 Jun 25;5(6):1018–33. doi: 10.1158/2767-9764.CRC-25-0024 (PMC12188421; doi:10.1158/2767-9764.CRC-25-0024)
Supplement: Supplementary Table S3 — Cdk1/2/5/9 gene expression differences between dinaciclib-resistant CCC organoids (19-055, 19-076, and 19-079) vs. the dinaciclib-sensitive CCC organoids (180-015, 19-001, 19-010, 19-042, 19-044, 18-148). A log fold change (logFC) greater than 0 was considered to indicate higher gene expression in dinaciclib-resistant samples. [file crc-25-0024_supplementary_table_s3_suppst3.docx]

| Gene | p-value | logFC |
| --- | --- | --- |
| *Cdk1* | 0.04213974 | -1.5327077 |
| *Cdk2* | 0.11966186 | -0.5794882 |
| *Cdk5* | 0.2766983 | -0.4463872 |
| *Cdk9* | 0.45977539 | -0.2437848 |

**Supplementary Table S3**

Cdk1/2/5/9 gene expression differences between dinaciclib-resistant CCC organoids (19-055, 19-076, and 19-079) *vs*. the dinaciclib-sensitive CCC organoids (180-015, 19-001, 19-010, 19-042, 19-044, 18-148). A log fold change (logFC) greater than 0 was considered to indicate higher gene expression in dinaciclib-resistant samples.
